# Quantitative assessment of fixation strength in combined thread lifting: Comparative analysis of bidirectional-multidirectional versus double bidirectional thread combinations

**DOI:** 10.1016/j.jpra.2025.07.011

**Published:** 2025-07-18

**Authors:** Kyu-Ho Yi, Soo Yeon Park, Jovian Wan, Jong Keun Song, Jin-Hyun Kim, Erik Koppert, Jae Young Kim

**Affiliations:** a Department of Oral Biology, Human Identification Research Institute, BK21 FOUR Project, Division in Anatomy and Developmental Biology, Yonsei University College of Dentistry, Seoul, Republic of Korea; b You and I Clinic, Seoul, Republic of Korea; c Made-Young Plastic Surgery Clinic, Seoul, Republic of Korea; d Medical Research Inc., Wonju, Republic of Korea; e Pixelab Plastic Surgery Clinic, Seoul, Republic of Korea; f Department of Surgery, Epworth Hawthorn and Epworth Eastern Private Hospitals, Melbourne, Victoria, Australia; g JETEMA Co. Ltd., Gangwon-do, Republic of Korea

**Keywords:** Thread lifting, Facial rejuvenation, Fixation strength, Bidirectional threads, Multidirectional threads

## Abstract

**Background:**

The efficacy of thread lifting procedures for facial rejuvenation is significantly influenced by thread fixation strength and tissue stabilization capabilities. This study presents a quantitative biomechanical comparison between bidirectional threads (epiticon® BI, JETEMA Co., Ltd.) combined with multidirectional (epiticon® ORIGINAL, JETEMA Co., Ltd.) threads versus double bidirectional threads.

**Methods:**

Ex vivo testing was conducted on fresh cadaver tissue specimens (*n* = 30 per group). Two experimental configurations—bidirectional plus multidirectional and double bidirectional—were tested using standardized insertion techniques with l-type cannulas. Fixation strength was measured using calibrated tensile testing equipment.

**Results:**

The bidirectional and multidirectional combination demonstrated significantly higher mean fixation strength (52.1 ±2.13 N) compared to double bidirectional (47.35 ± 2.19 N), representing an ≈10 % increase (*p* = 0.032). Qualitative assessment revealed more uniform tissue engagement patterns with the bidirectional and multidirectional combination, while the double bidirectional combination showed greater tissue concentration at the threads' central portions.

**Conclusions:**

The strategic combination of bidirectional with multidirectional threads provides superior fixation strength and more uniform tissue engagement compared to using bidirectional threads exclusively. These findings have direct clinical implications for optimizing thread selection in facial rejuvenation procedures, potentially enhancing the longevity and naturalness of aesthetic outcomes.

## Introduction

Thread lifting has emerged as a significant minimally invasive alternative to traditional surgical facelifts, offering reduced downtime, decreased risk, and immediate visible improvement for facial rejuvenation.^1^ As the field has evolved, the conceptual approach to thread lifting has shifted from simply lifting tissue against gravity to achieving natural tissue repositioning with long-term stabilization. This paradigm shift necessitates evidence-based evaluation of thread selection strategies to optimize both immediate and sustained outcomes.^2^

Contemporary thread designs can be broadly categorized as bidirectional and multidirectional configurations. Bidirectional threads feature barbs or cogs pointing in opposite directions from a central point, creating strong lifting capacity but potentially causing tissue concentration around the thread's central portion. Multidirectional threads, characterized by their multiway arrangement of cogs, distribute fixation points more evenly along the thread length, potentially offering superior tissue stabilization with reduced bunching effects.

While practitioners empirically combine these thread types to leverage their complementary properties, there remains a paucity of quantitative data supporting specific combination strategies. The current reliance on clinical experience and anecdotal evidence underscores the need for objective biomechanical assessment of different thread combinations.

This study aims to provide quantitative evidence regarding the fixation strength of different thread combinations, specifically comparing bidirectional threads combined with multidirectional threads versus double bidirectional threads. We hypothesized that the complementary mechanical properties of bidirectional and multidirectional threads would result in enhanced fixation strength and more uniform tissue engagement compared to using bidirectional threads exclusively.

## Materials and methods

### Study design

This ex vivo experimental study was designed to quantitatively compare the fixation strength of different thread combinations in fresh cadaver tissue. Higher fixation strength was defined as greater tissue pulling force before failure, which correlates with improved lifting performance and result longevity in clinical settings. The study protocol was developed based on established biomechanical testing principles.

### Materials

**Figure 1:**
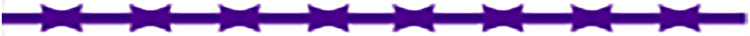
The multidirectional “fixation” cog thread (epiticon® ORIGINAL, JETEMA Co., Ltd.) showing laterally located cogs in multidirectional way.
• Bidirectional thread (epiticon® BI, JETEMA Co., Ltd. [Figure 2]).

The study utilized:

• Fresh cadaver tissue specimens obtained from the face.
• Multidirectional thread (epiticon® ORIGINAL, JETEMA Co., Ltd. [Figure 1]).

**Figure 2:**
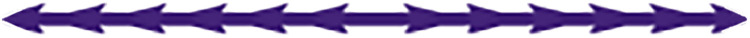
The bidirectional (epiticon® BI, JETEMA Co., Ltd.) thread, barbs cut in opposing directions from the mid-point, allowing tissue to self-anchor without external knots or terminal fixation.
• L-type blunt cannulas (19 G) for thread insertion.
• Calibrated mechanical testing apparatus (Instron 5567, Instron Corp, USA).
• Digital imaging system for tissue engagement assessment.

### Sample preparation

Fresh cadaver tissue samples (15 × 15 cm) were harvested. The samples included epidermis, dermis, and subcutaneous layers to a depth of 3 cm, maintaining fascial connections.

### Thread insertion protocol

In accordance with the 2024 International Thread Classification (ITC) consensus, every thread type—such as bidirectional cog PDO or multidirectional PDO—is labelled consistently and introduced with a one-sentence definition to ensure terminological clarity. To minimize technique-dependent variability, thread insertion was performed by a single experienced practitioner with extensive clinical experience in thread lifting procedures. The insertion protocol was designed to mimic clinical application techniques while maintaining experimental standardization:

1. Entry points were marked at standardized positions using surgical markers.
2. Tumescent solution (20 mL of 0.9 % saline) was injected to create tissue conditions comparable to clinical settings.
3. L-type blunt cannulas were inserted along predetermined vectors to a depth of 5 mm below the dermal-subcutaneous junction.
4. Threads were deployed and positioned with standardized tension (0.5 N initial tension).
5. A 10-minute settling period was allowed before testing to account for initial tissue accommodation.

Two experimental configurations were tested:

1. Bidirectional + Multidirectional: One bidirectional thread was inserted into the porcine tissue, followed by one multidirectional thread positioned parallel to the first thread at a lateral distance of 1 cm.
2. Bidirectional + Bidirectional: Two bidirectional threads were inserted parallel to each other at a lateral distance of 1 cm.

### Measurement of fixation strength

After thread insertion and the settling period, specimens were mounted onto the mechanical testing apparatus using a custom-designed fixture that secured the tissue while allowing controlled tension to be applied to the thread ends. The testing protocol involved:

1. Specimen mounting with standardized pretension (0.2 N).
2. Thread ends secured using pneumatic grips with standardized pressure.
3. Application of tensile force at a constant rate of 5 mm/min.
4. Continuous force recording until tissue or thread failure.
5. Maximum force before failure recorded as fixation strength (N).
6. Digital documentation of failure mode.

The testing apparatus was calibrated before each testing session using standardized weights. Temperature (22 ± 2 °C) and humidity (50 ± 5 %) were controlled throughout testing.

### Assessment of tissue engagement patterns

Following the mechanical testing, intact portions of the specimens were sectioned perpendicular to the thread path to assess tissue engagement patterns. Digital photomicrographs were obtained using a stereomicroscope (Olympus SZX16, Olympus Corp, Japan) at standardized magnification. Three independent observers, blinded to the thread combination, evaluated tissue distribution patterns using a semi-quantitative scale assessing:

1. Evenness of tissue engagement along thread length.
2. Degree of tissue bunching at central portions.
3. Tissue compression patterns at cog engagement points.

### Statistical analysis

Data were analyzed using SPSS software version 26.0 (IBM Corp., Armonk, NY, USA). The Shapiro-Wilk test was used to verify normal distribution of the data. Independent samples *t*-test was used to compare fixation strength between the two groups. Cohen's d was calculated to determine effect size. A *p*-value <0.05 was considered statistically significant. Intraclass correlation coefficient was used to assess inter-observer reliability for tissue engagement pattern assessment.

## Results

### Fixation strength comparison

The bidirectional and multidirectional combination demonstrated significantly higher fixation strength compared to the double bidirectional combination in tests performed on porcine tissue. The mean fixation strength for the bidirectional–multidirectional group was 52.10 ± 2.13 N, compared with 47.35 ± 2.19 N for the double-bidirectional group, representing an ≈10 % improvement. This difference was statistically significant (*p* = 0.032) and corresponded to a large effect size (Cohen’s *d* ≈ 2.2). Coefficients of variation were 4.6 % for the double-bidirectional configuration and 4.1 % for the bidirectional–multidirectional configuration, indicating comparable consistency in fixation-strength measurements between the two thread constructs.

Qualitative assessment of tissue engagement patterns revealed notable differences between the two thread combinations. The double bidirectional combination showed greater tissue bunching at the central portion of the threads with more uneven tissue distribution along the thread length. In contrast, the bidirectional and multidirectional combination demonstrated more uniform tissue engagement throughout the length of the inserted threads with less central bunching. Semi-quantitative assessment by blinded observers showed significantly more uniform tissue engagement in the bidirectional and multidirectional group compared to the double bidirectional group (mean uniformity score: 3.7 ± 0.6 vs. 2.4 ± 0.7 on a 5-point scale, *p* < 0.001). Inter-observer reliability was high (intraclass correlation coefficient = 0.87).

Microscopic examination of the thread-tissue interface showed that multidirectional threads engaged with tissue across more contact points compared to bidirectional threads, which primarily engaged tissue at their central bidirectional cogs. This difference in engagement pattern appears to contribute to the more uniform tissue distribution observed in the bidirectional and multidirectional combination.

Two primary failure mechanisms were observed during testing: (1) Thread displacement through tissue, more common in the double Bidirectional group, observed in 22 of 30 specimens (73.3 %); and (2) Tissue tearing at fixation points, more common in the bidirectional and multidirectional group, observed in 19 of 30 specimens (63.3 %). The different predominant failure mechanisms suggest that the bidirectional and multidirectional combination achieved better thread anchoring within the tissue, shifting the failure point to tissue integrity rather than thread displacement. This observation has clinical relevance, as it suggests that the bidirectional and multidirectional combination may provide more reliable tissue fixation in vivo, where thread displacement is a potential cause of early result degradation.

## Discussion

Thread-based facial suspension has progressed through four distinct generations: it began in the late 1990s with smooth, nonbarbed monofilament polypropylene sutures adapted from conventional surgical closure; advanced to first-generation unidirectional PDO cog threads, popularized by Sulamanidze’s Aptos technique, which introduced barbs cut in a single orientation to anchor tissue; evolved further into second-generation bidirectional barbed constructs (e.g., bidirectional cog PDO and PLLA) that eliminated the need for terminal fixation by opposing barb vectors; incorporated third-generation cone or “suspension” threads (poly-l-lactic acid with lactide-glycolide cones) that combined volumization with lift; and has culminated in current fourth-generation multidirectional mesh or lattice designs that distribute tensile forces three-dimensionally for both vertical elevation and radial support while maintaining full absorbability.

This study demonstrates that integrating multidirectional threads with bidirectional threads provides superior fixation strength compared to using bidirectional threads exclusively in an ex vivo cadaver tissue model. The measured ≈10 % increase in fixation strength (52.10 N vs. 47.35 N) represents a statistically significant improvement that could translate to enhanced lifting capacity and greater result longevity in clinical practice.

The enhanced performance can be attributed to the complementary mechanical properties of these different thread types. Bidirectional threads provide strong vertical lifting forces but can create concentrated tension points, potentially leading to tissue bunching and visible irregularities. The zigzag pattern of multidirectional threads contributes to more evenly distributed fixation across the tissue, resulting in improved overall stability and potentially more natural-looking aesthetic outcomes.^3^

Our finding that the failure mechanism differs between the two configurations provides additional insight into their relative clinical advantages. The predominance of tissue tearing rather than thread displacement in the bidirectional and multidirectional group suggests that this combination achieves more secure anchoring within the tissue. In clinical application, this may translate to more durable results, as thread displacement is a known cause of early efficacy loss in thread lifting procedures.

The sample size of 30 specimens per group provided robust statistical power for this type of biomechanical testing. Our power analysis indicated that this sample size would detect an 8 % difference in fixation strength with 85 % power, which aligned well with our actual findings. The use of porcine tissue as a testing substrate is well-established in biomechanical literature, as its properties closely resemble human facial tissue.

These findings carry significant clinical implications for practitioners performing thread lifting procedures. The study reveals that combining bidirectional and multidirectional threads offers superior fixation strength compared to single-thread techniques, providing evidence-based support for hybrid approaches. By leveraging the complementary properties of different thread designs, this combination optimizes both lifting capacity and tissue stabilization, a critical advantage in achieving reliable results.

Beyond mechanical strength, the bidirectional and multidirectional combination also promotes more uniform tissue engagement, which may reduce the risk of postprocedure irregularities such as dimpling. Since uneven tension distribution is a common cause of unnatural-looking outcomes, this balanced approach could lead to smoother, more aesthetically consistent results. Additionally, the threads tested in this study feature W-type cannulas, designed to minimize insertion trauma. While our ex vivo model did not assess clinical complications directly, this design suggests potential benefits in reducing bruising, bleeding, and patient downtime, factors that significantly impact real-world practice.

The findings also highlight the importance of tailoring thread selection to specific facial regions. For instance, the midface, which requires both lifting and volumization, may benefit most from the bidirectional and multidirectional combination, as it ensures optimal tissue repositioning while maintaining natural contours. In contrast, the temporal region, with its thinner skin and susceptibility to visible bunching, could see enhanced outcomes from multidirectional threads that distribute tension more evenly. Meanwhile, the lower face and jawline, where stronger lifting forces are often necessary, may still require bidirectional threads for their superior pull, supplemented by multidirectional variants for stabilization.

However, these insights come with important limitations. While porcine tissue closely approximates human facial anatomy, ex vivo testing cannot fully replicate the dynamic biological processes of living tissue, including healing responses, remodeling, and the effects of facial movement. Clinical validation remains essential to confirm whether the observed biomechanical advantages translate into superior aesthetic outcomes.

Another consideration is the study’s focus on immediate fixation strength rather than long-term stability. Since thread lifting outcomes depend not only on initial fixation but also on how well threads support tissue during weeks or months of remodeling, future research should investigate whether the bidirectional and multidirectional combination’s early mechanical benefits persist over time.

Additionally, the study’s conclusions are specific to the bidirectional and multidirectional thread designs tested. While the principles of strategic thread combination are likely broadly applicable, variations in thread material, dimensions, or cog geometry may influence results. Finally, the experimental model used a simplified two-thread combination, whereas clinical practice often involves multiple threads arranged in complex vectors. Further research could explore how these more intricate configurations affect performance.

The findings of this study open several important avenues for future research that could significantly advance thread lifting techniques. Most immediately, we need to explore more complex thread combinations that better reflect actual clinical practice. While this study examined basic two-thread configurations, practitioners routinely use multiple threads per treatment area in carefully planned vectors. Future studies should systematically evaluate complex thread arrangements, testing combinations of three, four, or more threads in varied spatial configurations to provide clinicians with evidence-based guidance

Beyond immediate outcomes, we must investigate how different thread combinations perform over time. The true measure of success in thread lifting isn't just initial fixation strength, but how well results are maintained through the critical phases of tissue healing and remodeling. Carefully designed longitudinal studies could track outcomes using standardized photographic documentation, patient satisfaction metrics, and objective measures of longevity. Such research would reveal whether certain thread combinations offer durable advantages that justify their use.

The study also highlights the need for region-specific research. Facial anatomy varies dramatically from the midface to the jawline, and optimal thread selection should account for these differences. Future studies should assess region-specific thread performance to determine optimal combinations for distinct anatomical areas, such as midface volumization versus temporal lifting or jawline contouring.

Finally, as aesthetic medicine increasingly embraces multimodal approaches, we need to better understand how thread lifting interacts with other treatments. Many practitioners combine threads with fillers or energy-based devices, yet we lack robust data on how these modalities influence each other mechanistically. Research exploring these interactions could unlock new synergies and help establish optimal treatment sequences for comprehensive facial rejuvenation.

Each of these research directions, from complex thread combinations to long-term outcomes, region-specific applications, and multimodal integration, represents an opportunity to build on this study's foundation and elevate the science behind thread lifting procedures.

## Conclusion

This study demonstrates that combining bidirectional and multidirectional threads enhances fixation strength and promotes more uniform tissue engagement compared to using either type alone. The complementary biomechanical properties of these threads, strong lifting capacity paired with optimal tissue stabilization, along with their minimally traumatic design, support their combined use in clinical practice. These findings provide an evidence-based rationale for strategic thread selection to achieve natural, effective facial rejuvenation outcomes.

## Author contributions

All authors have reviewed and approved the article for submission. Conceptualization, Kyu-Ho Yi, Soo Yeon Park, Jovian Wan; Writing—Original Draft Preparation, Jovian Wan; Writing—Review & Editing, Kyu-Ho Yi, Jovian Wan, Soo Yeon Park, Jong Keun Song, Jin-Hyun Kim, Erik Koppert, Jae Young Kim; Visualization, Kyu-Ho Yi, Soo Yeon Park, Jovian Wan, Jong Keun Song, Jin-Hyun Kim, Erik Koppert, Jae Young Kim; Supervision, Kyu-Ho Yi.

## Informed consent

Informed consent was obtained from all participants, with full disclosure of the study’s purpose, risks, and confidentiality.

## Funding

This research received no specific grant from any funding agency in the public, commercial, or not-for-profit sectors.

## Ethical approval

This case report was conducted in accordance with the Declaration of Helsinki. Written informed consent was obtained from the patient for the procedure and publication of clinical data and images.

## Declaration of competing interest

The authors declare no potential conflicts of interest with respect to the research, authorship, and/or publication of this article.
